# Cryo-EM structures of the immunodominant chlamydial antigen Major Outer Membrane Protein (MOMP) and its complex with a conformational neutralizing monoclonal antibody

**DOI:** 10.1063/4.0001131

**Published:** 2025-10-27

**Authors:** Yirui Guo, Megan Shelby, Patrik D'haeseleer, Sukumar Pal, Anatoli Slepenkin, Beverly Robinson, Zbyszek Otwinowski, Brent Segelke, Matthew Coleman, Dominika Borek, Luis de la Maza

**Affiliations:** 1 UT Southwestern, Dallas, TX, USA; 2 Ligo Analytics, Dallas, TX, USA; 3 Lawrence Livermore National Laboratory, Livermore, CA, USA; 4 UC Irvine, Irvine, CA, USA; 5 UC Davis, Sacramento, CA, USA

## Abstract

To understand immunogenicity of chlamydial Major Outer Membrane Protein (MOMP), we determined the structures of MOMP from Chlamydia muridarum elementary bodies and its complex with a Fab fragment of a neutralizing monoclonal antibody.

Natively folded MOMP forms a trimer of 10-stranded β-barrel protomers, with their extracellular regions folded into a single, compact magnesium- binding cap that contains immunogenic variable domains (VDs) from all three protomers. Sphingolipid molecules bound at the barrel interfaces further stabilize the trimer. The extracellular cap features positively charged cavities that may bind chlamydial and host molecules. The periplasmic region contains conserved cysteine residues, consistent with MOMP’s role in stabilizing the chlamydial outer membrane complex (COMC) through intramolecular disulfide bonds with other MOMP molecules and COMC proteins.

The β-barrels’ channels are too narrow to transfer previously characterized substrates, and are blocked by the extracellular cap and their N- terminal segments on the periplasmic side. Thus, native EB MOMP cannot function as a porin, suggesting that alternative MOMP folds may exist during intermediate and reticulate body stages, or that other proteins may perform this role.

In the MOMP-Fab complex (3:3 stoichiometry), each Fab binds two MOMP protomers, inducing structural changes in VD1 and VD4, which partially opens the antigenic cap without exposing the species-specific motif TTWNPTISG (TTLNPTIAG in C. trachomatis).

These findings challenge long-standing assumptions about MOMP’s architecture and open new avenues for research into MOMP’s role in chlamydial pathogenesis and development of therapeutics.

